# A predominant role of genotypic variation in both expression of sperm competition genes and paternity success in *Drosophila melanogaster*

**DOI:** 10.1098/rspb.2023.1715

**Published:** 2023-09-20

**Authors:** Bahar Patlar, Lauren Fulham, Alberto Civetta

**Affiliations:** Department of Biology, University of Winnipeg, Winnipeg, Manitoba, Canada R3B 2E9

**Keywords:** sperm competition, seminal fluid, GWAS, genotype-by-environment interactions, gene expression, *cis* regulation

## Abstract

Sperm competition is a crucial aspect of male reproductive success in many species, including *Drosophila melanogaster,* and seminal fluid proteins (Sfps) can influence sperm competitiveness. However, the combined effect of environmental and genotypic variation on sperm competition gene expression remains poorly understood. Here, we used *Drosophila* Genetic Reference Panel (DGRP) inbred lines and manipulated developmental population density (i.e. larval density) to test the effects of genotype, environment and genotype-by-environment interactions (GEI) on the expression of the known sperm competition genes *Sex Peptide*, *Acp36DE* and *CG9997*. High larval density resulted in reduced adult body size, but expression of sperm competition genes remained unaffected. Furthermore, we found no significant GEI but genotypic effects in the expression of *SP* and *Acp36DE*. Our results also revealed GEI for relative competitive paternity success (second male paternity; P2), with genes’ expression positively correlated with P2. Given the effect of genotype on the expression of genes, we conducted a genome-wide association study (GWAS) and identified polymorphisms in putative *cis*-regulatory elements as predominant factors regulating the expression of *SP* and *Acp36DE*. The association of genotypic variation with sperm competition outcomes, and the resilience of sperm competition genes’ expression against environmental challenges, demonstrates the importance of genome variation background in reproductive fitness.

## 1. Introduction

Males typically transfer several types of seminal fluid proteins and peptides (Sfps) along with the sperm in their ejaculate. Sfps have a diverse array of functions that can help reduce the risk and intensity of sperm competition [1,2]. For example, some Sfps can reduce the remating rate of females by altering their mating behaviour or physiology [3–6], contribute to the formation of mating plugs that can physically block the genital organs after receipt of an ejaculate [7,8], or aid sperm entrance into storage and its retention [9–12].

Given their role in reproductive fitness and their pattern of rapid evolution among species [13–16], it is commonly assumed that most Sfps have evolved under sexual selection driving species-specific adaptations. However, the effectiveness of post-mating sexual selection acting upon Sfp-producing genes can be reduced due to male-biased expression and limited genetic variance in sperm competing within females [17–19]. Interestingly, we have recently found in *Drosophila melanogaster* that many Sfp-producing genes, including sperm competition genes, diverge rapidly but exhibit high levels of polymorphism, signalling a relaxation of selection [20]. We have suggested that while selection is predominantly relaxed at the level of coding Sfp gene sequences, regulation of Sfp gene expression may be subject to strong selection, especially in competitive environments [20].

In *D. melanogaster*, the importance of Sfp expression on male competitive fitness has been shown by the use of gene-specific knockdowns and knockouts [21–24], which often examined the effects of a lack or near lack of gene expression. However, the genotypic variation in gene expression and its relationship to fitness has surprisingly only been addressed to a limited extent [25,26]*.* Furthermore, Sfp abundance and the expression of Sfp genes are plastically adjusted in many taxa, including *D. melanogaster*, depending on the risk and intensity of sperm competition [27]. The majority of studies have examined changes in Sfps depending on changes in perceived risk of sperm competition by manipulating population size at development (e.g. larval density) or adult stage and showed average directional effects of either decrease or increase of Sfp gene expression when exposed to rivals [28–32]. However, these studies often used samples from single wild-derived stocks and did not measure individual differences among environments *per se*, thus the extent of variation in genotypic response that is driven by genotype-by-environment interactions (GEI) remains largely underexplored. To our knowledge, only a couple of studies have examined the effect of GEI on the expression of a number of Sfp genes that included some with known roles in sperm competition [25,33]. Moreover, there is a significant lack of studies mapping genetic polymorphisms contributing to variation in gene expression of Sfps [34].

The evolution of sperm competition genes is likely dependent on different ecological variables. Conditions such as developmental population density are thought to be a factor influencing male perception of the risk of sperm competition, thus affecting male investment in reproduction [35–38]. In *D. melanogaster*, the number of larvae per nest site (i.e. larval density) varies in nature, affecting resource availability [39,40] and risk of sperm competition [41]. In this study, we therefore investigated the effects of genotype, larval density and their interaction (GEI) on variation in the expression of sperm competition genes in *D. melanogaster*. To do so, we selected three genes: *Sex Peptide* (*SP*), *Acp36DE* and *CG9997*, which are functionally well studied, and known to affect sperm competition [24], and measured the variation in their gene expression by exposing inbred lines from the *Drosophila* Genetic Reference Panel (DGRP) to low and high larval densities. We also tested for correlations between gene expression and relative paternity success in competition using DGRP lines. In addition to our experiments, we used the published genome and transcriptome data of the DGRP lines [42,43] to identify genome-wide polymorphisms associated with variation in the expression of our target genes.

## 2. Material and methods

### (a) Fly stocks and maintenance

Most fly strains were obtained from the Bloomington *Drosophila* Stock Centre (BDSC), Bloomington, IN, USA. We used 34 inbred lines from the *Drosophila* Genetic Reference Panel (DGRP) [42] for gene expression measurements and sperm competition experiments. To assess relative paternity success of DGRP males in sperm competition, we competed them against *D. melanogaster* males that express the green fluorescent protein (GFP) in their eyes and ocelli (BDSC32175). Females used were from a strain of *D. melanogaster* derived from a single female (an isofemale line) captured in Winnipeg in 2018 (Wpg02). All strains were kept as non-overlapping generations at 22°C under 12 : 12 h light-dark cycles in vials or bottles filled ad libitum with standard cornmeal-yeast-molasses-agar (CYMA) fly medium.

### (b) Larval density treatment

To study how population density during development affects the expression of *Acp36DE*, *CG9997* and *SP*, we manipulated larval density of each DGRP line. We started by collecting approximately 200 males and 200 females from each DGRP line within 3 h of hatching. Males and females were separated and kept in bottles containing CYMA medium for three days to allow them to reach sexual maturity. Afterwards, males and females were brought together in equal numbers into two egg chambers (i.e. two replicates) with Petri dishes containing a grape juice/agar medium and allowed to mate and lay eggs overnight. The next day, we collected 600 eggs from the surface of the grape medium and randomly divided the eggs into vials or bottles to create high and low larval density environments. For each DGRP line and replicate, we set up two 36 ml vials with 4–5 ml of standard fly medium, each with 200 eggs, and two 250 ml bottles with 50 ml of standard fly food, each with 100 eggs. In total, we created 4 high (vials) and 4 low (bottles) treatment replicates for each DGRP line. To control for the effects of age and mating status on gene expression [44,45], we monitored the vials and bottles continuously while the offspring hatched and collected the male offspring within 3 h of hatching. For three days, we placed the males in vials at a constant density of five adults per vial with ad libitum food.

A subset of 3 day old males (*n* = 10) was randomly chosen from each DGRP line and larval density condition to test the effect of treatment on body size. The right wing of each male was removed and mounted using 10 µl of mounting solution (70% glycerol and 30% ethanol). Images of wings were digitally captured using a binocular microscope with 10× magnification. We measured the centroid size of the wing as a proxy for body size [46]. We followed the landmarking procedure [47] using *tps* software [48].

### (c) Gene expression measurements

We used a subset of males from the larval density treatments to measure *SP*, *Acp36DE* and *CG9997* expression. We dissected male reproductive tracts (testes, accessory glands and ejaculatory bulbs) from five randomly chosen males from each environment and DGRP line replicates. We then isolated total RNA using the Bio-Rad Aurum Total RNA Mini Kit (Bio-Rad, CA, USA) and subsequently synthesized complementary DNA (cDNA) using 1 µl RNA solution and the iScript Select cDNA Synthesis Kit (Bio-Rad, CA, USA) following the manufacturer's instructions. In total, we obtained four cDNA biological replicates for each DGRP line and larval density treatment.

The expression of three genes from 272 samples (34 lines × 4 replicates × 2 treatments) were quantified using the Quant Studio 3 Real-Time PCR 384-well system (Thermo Fisher Scientific). The primers used were designed using Primer3Plus (http://primer3plus.com) and obtained from Integrated DNA Technologies (IDT), ON, Canada. The qPCR reactions were performed using the iQ SYBR Green Quantitative Real-Time PCR Kit (Bio-Rad, CA, USA). Reaction volumes were set at 12 µl and contained 4 µl iQ SYBR Green Superfix Kit (Cat. 1708880), 150 nM of each primer pair (1.5 µl per pair), 4 µl nuclease-free water and 1 µl cDNA. Thermal cycling conditions were 1 cycle at 95°C for 5 min, followed by 39 cycles of denaturation at 95°C for 15 s and annealing at 59°C for 30 s.

The absolute cycle quantification (*C*_q_) values of the genes were standardized by using a different gene in the same qPCR system, namely the ribosomal protein-coding gene *RpS18*, which was previously confirmed as a reliable housekeeping gene control [49]. The relative expression of the target genes was calculated by subtracting the *C*_q_ value of the reference gene from the *C*_q_ value of the target gene (i.e. Δ*C*_q_) [50]. These calculations were performed after verifying that the expression of *RpS18* was consistent between DGRP lines and density treatments (see statistical analysis section).

### (d) Sperm competition assay

We randomly selected a subset of 11 DGRP lines from our starting 34, and grew males from each of these lines under different larval densities using the protocol described above. We also grew reference males from the GFP eye-expressing strain and Wpg02 wild-type females under standard density conditions.

We collected virgin GFP males and females and kept them in sex-separated vials (10 individuals/vial) with ad libitum food for 3 days. Then, we paired GFP males and females randomly in groups of 20 couples in a bottle and left them for 24 h. The following day, we removed the males and individually placed females in fresh vials (vial 1). To ensure females became receptive to a second mating [51], females were left in vial 1 for 2 days. After 2 days, we transferred the females to a new vial (vial 2). Females in vial 2 were separated into two groups, with half being introduced to DGRP males that had been grown under low and the other half to DGRP males grown under high larval density conditions. We left the pairs to mate overnight and removed the males the next day. Three days after the second mating, we transferred the females to fresh vials (vial 3) and discarded them 4 days later. We counted the offspring from all the vials until the last fly hatched.

Lack of progeny in vial 1 (6 out of 368) allowed us to identify and discard females that did not receive sperm during the first mating. Females that did not produce wild-type offspring (149 out of 362) were excluded under the assumption that they did not mate to the second male. Therefore, we assess the relative success as net progeny production of each male and only used females that produced offspring from each of the mating guarantying the sperm mix and usage from each mate. The relative paternity success of the second male was calculated as the proportion of wild-type offspring to the total number of offspring (P2) over vials 2 and 3.

### (e) Genome-wide associations

We performed a Genome-Wide Association Study (GWAS) to identify DGRP sequence variants (SNP and non-SNP variants) associated with variation in *Acp36DE* and *SP* gene expression. We used DGRP Freeze 2.0 (http://dgrp2.gnets.ncsu.edu), which includes whole-genome sequencing data from 205 *D. melanogaster* lines along with genotype calls [42,43]. Unfortunately, the whole-genome sequencing data does not include *CG9997*, so we excluded this gene from our GWAS. Gene expression data for *SP* and *Acp36DE* were obtained from the *dgrp2* website, which contains gene expression data from adult males raised at 25°C for 185 DGRP lines [52,53]. We used gene expression as phenotypic data to run the GWAS tool available on the *dgrp2* website [43]. The location and gene annotation of significant positions were extracted from the DGRP variants’ annotations. In cases where multiple genes were associated with a single position, each gene was considered.

### (f) Statistical analyses

Statistical analyses were performed using R version 4.2.2 (R Development Core Team, 2014). We implemented Generalized Linear Mixed Models (GLMMs) that included replicates as a random factor to determine the effects of fixed factors, i.e. DGRP lines (i.e. genotypes), larval density treatments (i.e. environment) and their interactions on the phenotypes tested (i.e. body size, absolute gene expression of *RpS18* and the relative expression of sperm competition genes). We implemented GLMM with a binomial error distribution and a logit link function to test the main effects of genotype, environment and their interaction on paternity success in sperm competition assay. The model included the response variable as a matrix where the first column is the number of wild-type offspring and the second column is the number of GFP-expressing offspring, and random factors of female ID and treatment replicates. We used two-way Analyses of Variance (ANOVA) to test the relationship between paternity and gene expression using DGRP averages for gene expression and paternity success.

The coefficient of variation (CV) was used as a measure of the degree of variability in expression of each gene among DGRP genotypes in each larval density environment. We used the Modified Signed-Likelihood Ratio (MSLR) test in the R package of *cvequality* using standard 1000 simulations to analyse differences in the CV between larval density treatments [54,55]. Broad sense heritability (*H*^2^) of gene expression was estimated as the ratio of genotypic variation to the total phenotypic variation among DGRPs [56]. We tested whether the *H*^2^ estimates were significantly greater than zero using one-sided *z*-tests.

## 3. Results

### (a) Genotype-dependent effects on body size under different larval density conditions

**Figure 1.**
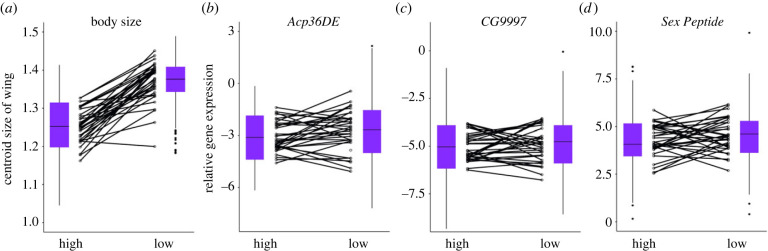
Plots showing the relationship between larval density and the response variables (*a*) body size and (*b–d*) relative expression of sperm competition genes. Boxplots represent the 1st and 3rd quartiles of the data, where lines are the medians, whiskers are the range of data and solid circles are data points that fall outside the 1.5 times interquartile range. Empty circles are average values for DGRP lines and black horizontal lines show the direction and degree of response of DGRP lines to different environments.

Overall, it is expected that body size should decrease at high density compared with low density due to less availability of resources per individual. However, the effect of density on body size could also be dependent on genotypes. Therefore, we tested the effects of genotype, environment and genotype-by-environment interaction on body size. We found that larval density dramatically altered the overall body size of DGRP lines, with the majority of lines being larger when reared at low densities (figure 1*a*). In addition, significant genotype and GEI effects were found (electronic supplementary material, table S1). The GEI identifies that some DGRP lines did not respond to differences in larval density rearing, as seen in the individual DGRP reaction lines (figure 1*a*).

### (b) Genotype, but not larval density, affects expression of Sfp genes

We first tested the consistency of absolute expression of the reference gene *RpS18* between the environments and the DGRP lines. We found that manipulation of larval density slightly affected the expression of *RpS18* (electronic supplementary material, table S2*a*, and figure S1*a*). Prior to the relative gene expression calculations, we performed an outlier analysis to detect samples that violated the consistency between environments for *RpS18* expression (electronic supplementary material, figure S1). To do this, we counted samples with an expression value 1.5 times the interquartile range above the third quartile or below the first quartile. We identified three individual samples as outliers (1.1% of the total data) and excluded them from the analysis of *RpS18* expression. After removing the outliers, we found that larval density had no dramatic effect on absolute *RpS18* expression (electronic supplementary material, table S2*b*, and figure S1*b*). The marginal effect is negligible, as we are dealing with more than a hundred samples per environment instead of a few samples that might react to a small shift in the mean. Subsequently, we calculated the relative expression of *SP*, *Acp36DE* and *CG9997* by removing *RpS18* outliers and fitted GLMMs that included the random factor of replicates and the fixed factors of genotype, environment and GEI. The results show that the relative expression of the three genes was not significantly affected by larval density treatments (table 1, figure 1*b–d*). We also did not observe significant GEI, although the reaction lines of the genotypes frequently crossed each other (figure 1*b–d*). To be certain that any marginal effect of environment on *RpS18* did not affect conclusions about the effect of treatment on relative Sfp gene expression, we also tested the effect of larval treatment on absolute Sfp expression. We found that the absolute expression of all Sfp genes is not affected by larval density, with or without outliers (electronic supplementary material table S3; and figure S2). We found a significant effect of genotype on the relative expression of *SP* and *Acp36DE*, with the genotype effect being stronger (*p* < 0.01) for the relative expression of *Acp36DE* (table 1).

**Table 1.** Generalized Linear Mixed Models for fixed effect terms showing significant genotype effects on gene expression. The factors include genotype (DGRP inbred lines), environment (larval density treatment), and GEI (genotype-by-environment interaction). Significant *p*-values < 0.05 are bolded. SS, sum of squares; MS, mean sum of squares.

	d.f.	SS	MS	*F* ratio	*p*-value
***Sex Peptide***					
environment	1	5.243	5.242	2.590	0.109
genotype	33	109.198	3.309	1.635	**0**.**022**
genotype × environment	33	70.468	2.135	1.055	0.395
***Acp36DE***					
environment	1	7.637	7.637	2.426	0.121
genotype	33	192.273	5.826	1.851	**0**.**006**
genotype × environment	31	62.894	1.965	0.624	0.943
***CG9997***					
environment	1	1.865	1.865	0.717	0.398
genotype	33	105.245	3.189	1.226	0.199
genotype × environment	32	56.693	1.718	0.660	0.921

We observed a decrease in variance among DGRP lines in the expression of *Acp36DE* and *CG9997* at high compared with low larval density (figure 1*b,c*). Therefore, we calculated the CVs and tested for equality of the estimates between environments. In line with our qualitative observation, the results of the MSLR test showed that variation in expression of *Acp36DE* and *CG9997* decreases significantly at high density (table 2). Interestingly, broad-sense heritability of gene expression per density environment is significant at low density for each of the genes, whereas this is not the case at high density (table 3).

**Table 2.** Modified Signed Log-Likelihood Ratio test shows significant differences in variation of gene expression across genotypes between high and low larval density treatments. CV = coefficient of variation. Significant *p*-values < 0.05 are bolded.

	CV (%) low density	CV (%) high density	MSLR	*p*-value
*Sex Peptide*	18.71	19.11	0.020	0.888
*Acp36DE*	41.65	28.31	105.861	**<0**.**05**
*CG9997*	18.19	14.20	169.563	**<0**.**05**

**Table 3.** Variance components (*V*_G_: genotypic variance, *V*_P_: phenotypic variance), broad heritability (*H*^2^) and standard error (SE) of broad sense heritability for gene expression in low and high density treatments. Bold *p*-values indicate *H*^2^ significantly different from zero.

	low density					high density				
	*V*_G_	*V*_P_	*H*^2^	SE	*p*-value	*V*_G_	*V*_P_	*H*^2^	SE	*p*-value
*Sex Peptide*	0.250	0.726	0.344	0.151	**0**.**01**	0.141	0.648	0.217	0.166	0.10
*Acp36DE*	0.345	1.312	0.263	0.160	**0**.**05**	0.130	0.709	0.183	0.168	0.14
*CG9997*	0.222	0.781	0.284	0.158	**0**.**04**	0	0.671	0	0.171	0.50

### (c) Sperm competitiveness is dependent on both genotype and larval density

We first analysed the relative paternity success of DGRP males, measured as P2, for genotype, environment and their interaction effects. The results showed that P2 changes significantly between DGRP lines, while larval density has no effect on average paternity success (electronic supplementary material, table S4). We also found that genotypes differ in their sperm competition success depending on the larval density environment they experience during development (i.e. GEI effect) (electronic supplementary material, table S4).

**Figure 2.**
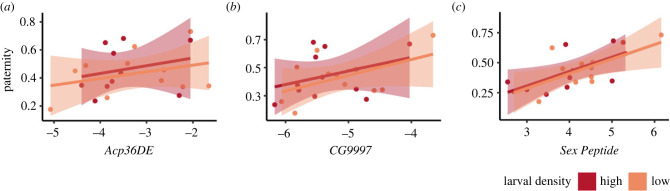
The relationship between seminal fluid expression of genes and relative paternity success (P2) of DGRP lines in low and high larval density treatments. The lines show trends in data response according to the linear regression models. Shaded areas show 95% confidence intervals.

Given the significant effects of genotype on competitive paternity success (P2), as well as in *SP* and *Acp36DE* expression, we examined the relationships between gene expression and P2 using DGRP line averages and including the larval density treatment as a covariate to avoid pseudo replication. The relationships between average P2 and sperm competition gene expression showed a similar pattern in each environment for each gene, with P2 increasing with increasing gene expression (figure 2). But the ANOVA statistics show that only the expression of *SP* is significantly correlated with paternity success (electronic supplementary material, table S5).

### (d) GWAS identifies a predominant role of *cis*-regulatory polymorphisms

Associations between sequence polymorphisms (i.e. SNPs, insertions, deletions) and gene expression data from 185 DGRP lines were performed using 1 891 697 and 1 896 742 polymorphic positions with minor allele frequency (MAF) higher than 5% in *SP* and *Acp36DE*, respectively. We applied False Discovery Rate (FDR) corrections, however, we only found two significant SNPs for *SP* expression at a cut-off of *p* < 0.05. Due to the existence of linkage among genome sites, correction methods such as Bonferroni or Benjamini–Hochberg FDR that assume independence among detected variants are overly stringent and can result in a high number of false negatives [57,58]. Here, we used a *p* < 10^–5^ cut-off, without FDR correction, that is commonly applied in GWAS using DGRP lines [59–61].

**Figure 3.**
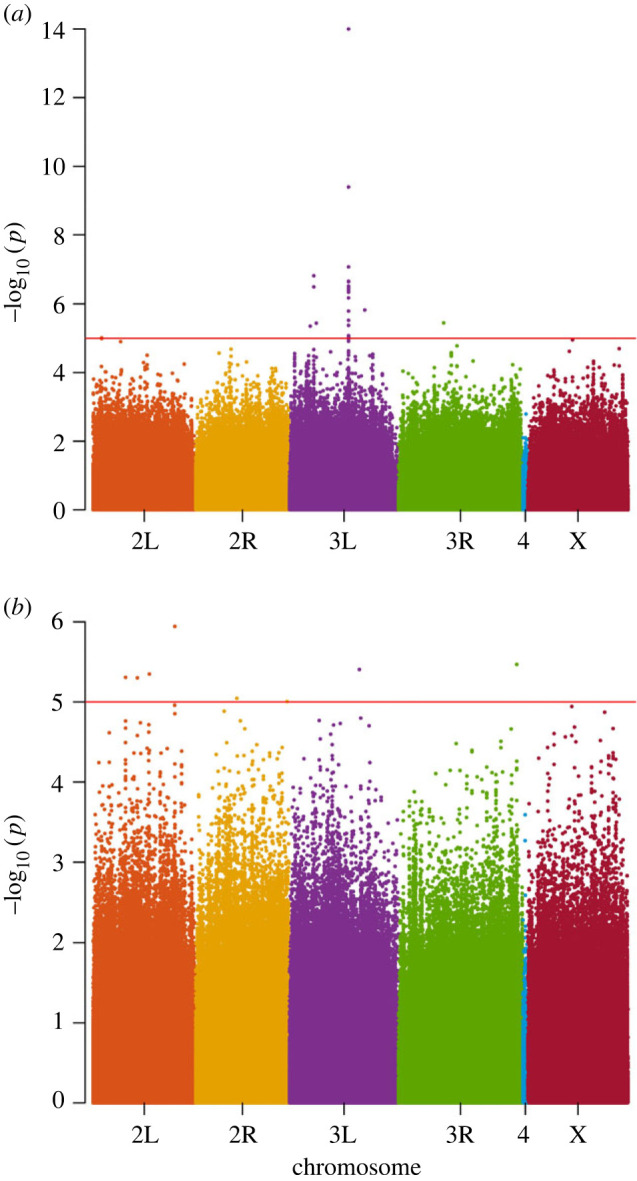
Genome-Wide Association Study (GWAS) between polymorphisms and gene expression. Manhattan plots of the results from the GWAS for (*a*) *Sex Peptide* and (*b*) *Acp36DE*. The red line corresponds to the critical *p*-value < 1 × 10^−5^.

We found 22 variants with effects on expression of *SP* and 8 variants with effects on *Acp36DE* expression (electronic supplementary material, table S6, figure 3). The location of variants affecting the expression of target genes could be within non-transcribed DNA-binding sites (e.g. promoter elements or enhancers) or within transcripts or proteins acting as regulatory elements (e.g. transcription factors). For *SP*, 91% (20 out of 22) of the variants associated with variation in expression were found in chromosome 3L, where *SP* maps, and the majority of those either upstream or downstream of the reading frame of annotated transcripts (i.e. outside untranslated regions (UTRs), exons, introns) (electronic supplementary material, table S6, figure 3). Moreover, 15 of the 22 variants (68%) mapped within an approximately 3.5 Kb region around *SP* itself (electronic supplementary material, figure S3). There were fewer variants affecting *Acp36DE* expression, with half of them located on the same chromosome arm as *Acp36DE* (2L) and most variants within the transcripts reading frames of the annotated genes (electronic supplementary material, table S6).

Only a few polymorphisms suggested changes in expression driven by changes within coding sequence of putative *trans*-regulatory elements. *SP* and *Acp36DE* expression were affected by polymorphisms within exon regions of two lncRNAs (*CR43911* for *SP* and *CR43238*) (electronic supplementary material, table S6). For *SP*, there was one non-synonymous polymorphism mapped within *SP* itself (Ala → Ser) and another within *CG6592* (Asn → Asp), a serine endopeptidase (https://flybase.org/) with a predicted DNA-binding domain (https://dnabind.szialab.org/). One nonsynonymous polymorphism (Leu → Val) within *CG5853* affected the expression of *Acp36DE*.

## 4. Discussion

Based on classical theories of sperm competition [62–64], high larval density is expected to lead to increasing ejaculate investment due to perceived sperm competition, which has been supported by empirical evidence of increasing sperm count or spermatophore size [65–67]. Increased larval density has also been shown to negatively affect body size and accessory gland size in *D. melanogaster* [68], and the effect on accessory gland size remained even when body size was held constant in all larval density treatments [38]. Our manipulation of larval density resulted in smaller adult males at high density, and we expected that the expression of Sfp genes should also be impacted by larval density conditions. However, we found that larval density treatment had no effect on the expression of the sperm competition genes *SP*, *Acp36DE* and *CG9997*. Furthermore, the genotypes did not show different patterns of gene expression depending on larval densities (i.e. non-significant GEI). Our findings of limited response to larval density manipulations are in agreement with other studies that showed no response to differences in larval density of the expression of *Acp36DE* [69] and SP abundance in *D. melanogaster* [29]. The broader surveys of the expression of Sfp genes under different larval density conditions done in nematodes have found no effects due to larval density manipulations [25,33]. There are different reasons why expression of sperm competition genes shows resilience against environmental challenges. It is possible that smaller males with smaller accessory glands increased seminal fluid gene transcription due to perceived competition or to maintain a required level of gene expression for proper tissue function, thus leading to no differences in expression in small males relative to larger males with larger glands. Alternatively, if smaller glands do not alter the number or size of cells responsible to produce Sfp transcripts, similarities in expression under different larval density conditions might truly reflect a lack of environmental effects in regulation. Future studies testing larval density effects will need to evaluate changes in the accessory gland cell content and assay expression pattern using single cell approaches rather than bulk quantifications. Taken together, our results cannot fully rule out regulation of Sfp gene expression as an insensitive trait to the organ size or the perceived risk of sperm competition via developmental conditions but demonstrate that expression of sperm competition genes is resilient against larval density conditions. It is worth mentioning that perceived sperm competition during development is likely to be a factor influencing the outcome of sperm competition. However, it is conditions in adulthood, such as the number of rivals and the duration of contact with rivals, that may have a stronger influence on male phenotypes in sperm competition. For example, copulation duration in *D. melanogaster* increases with increasing duration of contact with rivals, suggesting increased investment in ejaculate [70]. Moreover, males exposed to one or four males during the premating episode have shown no difference in the expression of some Sfps in the first 24 h, but do so after 72 h [28]. Since we kept our males in the same environment with the same density of adult males before the sperm competition assays, we can assume that the experimental males are standardized with respect to their adult environment. To date, little is known about the effects of potential interactions between developmental and adult environments in males on sperm competition phenotypes.

The significant genotypic effect on *SP* and *Acp36DE* expression is in agreement with a few studies that have shown high variation in Sfp expression between genotypes [25,26,71]. Although there is now increasing evidence of genetic background differences effects on Sfp expression, what evolutionary processes have led to this variance remains unclear. One possibility is that selection for expression of sperm competition genes is relaxed. As suggested earlier, only a small fraction of genetic variation actually competes in female reproductive tracts, reducing the effectiveness of selection [19]. Secondly, Sfps can be beneficial for rival ejaculates, for example, *SP* has the ability to bind to female-stored sperm from a previous mating partner, promoting the effective release and use of all stored sperm [72]. Therefore, a well-conditioned male Sfp composition can decrease the selection on rivals and maintain variation. Another plausible explanation for the maintenance of variance is the existence of significant GEI. GEI has been proposed to explain the maintenance of genetic variation in traits that are often subject to strong selection [18,73,74], and found to explain variation in the expression level of a sperm competition gene in *Macrostomum lignano* [33]. Here, we did not detect GEI for sperm competition expression of genes, however, we found that variance across genotypes at high density is lower for both *Acp36DE* and *CG9997*. A decrease in variance is expected under strong selection, which is likely in a high-density competitive environment. Moreover, we found GEI for relative paternity success in competition (P2), and the expression of sperm competition genes was positively correlated with P2, particularly for *SP*. Finally, given the non-transitive relation among genotypes in sperm competitiveness [75,76] and the correlation we observed between P2 and expression, non-transitivity might also help maintain variation in Sfp gene expression.

We have used a GWAS analysis to identify polymorphisms that associate with changes in the expression of sperm competition genes *SP* and *Acp36DE*. The differential expression of these genes could be driven by a variety of regulatory differences. Our analysis allows us to draw some conclusions about the possible nature of regulatory changes underlying changes in expression across genotypes. First, we find a very limited number of polymorphisms mapping within either transcribed or translated gene products (electronic supplementary material, table S5) suggesting a limited role of changes in coding sequence of putative *trans*-regulatory elements. The analysis of allele-specific expression using transcriptomics data have highlighted a main role of *trans*-regulatory changes driving variation in genome-wide expression within species [77–81]. Thus, regulation of variation in expression of *Acp36DE* and *SP* appears to deviate from the genome-wide pattern. Second, most polymorphisms mapped up- or downstream of annotated genes, indicating that changes in *cis*-regulatory elements (promoter/enhancers) are prevalent. We do not know whether polymorphisms in these non-coding positions directly affects *Acp36DE* and *SP* expression or whether the effect is mediated by changes in the expression of other genes that interact with *Acp36DE* and *SP*. An interesting observation is that several polymorphisms mapped nearby lncRNAs. lncRNAs are known to have functions in regulation of transcription [82,83] and our results suggest, at least for *SP*, a possible role of lncRNAs in the regulation of expression. While our GWAS did not include treatment effects on expression, lncRNAs have been suggested as modulators of transgenerational epigenetic inheritance of stress conditions [84,85]. Therefore, it will be worth testing the relation between *SP* expression and paternal effects on offspring phenotypes. Third, if the effect of the mapped polymorphisms in non-coding sequence regions is exerted upon different genes’ expression, rather than direct effects on our targets, the observation of a cluster of polymorphisms (electronic supplementary material, figure S1) suggests a common system of regulation of the different expression of genes. This type of compartmentalization is found in situations where a common regulatory element with a regional effect, or differential chromatin-based regulation, is at work [86,87]. Fourth, we found no evidence of polymorphism within or nearby other Sfps influencing the expression of *Acp36DE* and *SP*. Therefore, while epistatic interactions might be important for the function exerted by the proteins [88–91], we did not detect any evidence of epistasis at the level of regulation of gene expression among Sfp genes in *D. melanogaster*.

In conclusion, we showed that larval density treatment had no significant effect on the expression of sperm competition genes *SP*, *Acp36DE* and *CG9997*, and no effect on the response of the different genotypes. However, the expression of *SP* and *Acp36DE* was found to show significant genotypic variation between the DGRP lines, and GWAS analysis indicated that the changes in expression were likely due to changes in *cis*-regulatory elements (promoter/enhancer), with an interesting clustering of polymorphisms affecting the expression of *SP*.

## Data Availability

The input files of the data obtained from the study are deposited in Dryad Digital Repository: https://dx.doi.org/10.5061/dryad.j9kd51cj9 [92]. Supplementary material is available online [93].
